# Human primary proximal tubular epithelial cells and sepsis: a scoping review

**DOI:** 10.1007/s10157-025-02656-1

**Published:** 2025-03-26

**Authors:** Kjellbjørn Jakobsen, Ludvik K. Ellingsen, Eirik Reierth, Stephen J. Hodges, Lars M. Ytrebø

**Affiliations:** 1 Anesthesia and Critical Care Research Group, UiT-The Arctic University of Norway, Tromsø, Norway; 2 Division of Surgical Medicine and Intensive Care, University Hospital of North Norway, Tromsø, Norway; 3 University Library, Science and Health Library, UiT-The Arctic University of Norway, Tromsø, Norway; 4 CSO Haoma Medica LtdHaoma Medica Ltd, London, United Kingdom

**Keywords:** Human proximal tubule cells, Sepsis, Acute kidney injury, In vitro, Experiment

## Abstract

**Background:**

Sepsis impairs proximal renal tubular epithelial cell (PTEC) function, and this impairment contributes to the pathophysiology of sepsis-associated acute kidney injury (SA-AKI). By closely replicating in vivo conditions, primary human PTEC offer superior biological relevance for studying SA-AKI. The purpose of this scoping review was to identify and investigate experiments, where human primary PTEC have been used to study sepsis-related factors.

**Methods:**

A comprehensive literature search strategy was developed, and our reported items adhered to the Preferred Reporting Items for Systematic Reviews and Meta-Analyses extension for scoping reviews (PRISMA–ScR) checklist. Peer-reviewed articles published in English or Scandinavian languages between 1946 and 2024 were included.

**Results:**

The literature search provided 292 results. Twelve studies were included, out of which only two were published after 2010. Eight studies used human primary PTEC isolated from healthy tissue during tumor nephrectomy, while four studies used primary PTEC purchased from commercially available providers. Experimental methods were heterogenous. The included studies applied *E. coli* porine, *E. coli*, Staphylococcal enterotoxin B, cytokines and lipopolysaccharide with differing dosages, exposure lengths, and combinations.

**Conclusions:**

Although human primary PTEC more closely resembles the in vivo environment in human kidneys, their use in sepsis and SA-AKI research remains remarkably limited, leading to substantial research gaps in the field. In addition, there is significant heterogeneity in the methodologies employed across existing studies. Standardizing and expanding the use of primary PTECs in future in vitro research could be pivotal for unraveling novel and relevant insights into the pathophysiology of SA-AKI.

## Introduction

Sepsis leads to life-threatening organ dysfunction caused by a dysregulated host response to infection [1]. Sepsis is a common feature in patients treated in critical care units and sepsis-associated acute kidney injury (SA-AKI) develops in 30–60% of the patients [2]. SA-AKI is associated with acute mortality, and poor outcome persists despite high clinical vigilance and implementation of various critical care supportive measures [2]. The precise pathophysiological mechanisms underlying SA-AKI remain incompletely delineated [3]. Sepsis can impair proximal renal tubular epithelial cell (PTEC) function, and this is considered to contribute to SA-AKI pathophysiology [3]. Metabolic dysregulation has emerged as a key factor, particularly regarding its impact on PTEC. During sepsis, mitochondrial dysfunction and impaired fatty acid oxidation in PTEC can lead to substantial cell damage, contributing to both acute and chronic kidney dysfunction [4]. This metabolic failure is often described as a breakdown of the starvation response, in which essential metabolic pathways deteriorate and exacerbate damage during sepsis [5].

Human primary PTEC are available from human renal tissue at the time of biopsy or may be purchased from commercially available providers. Human primary PTEC can be propagated in monoculture for a limited period and studied before evidence of senescence occurs [6].

Established kidney cell lines are extensively used for the study of SA-AKI due to their ease of use, cost-effectiveness and the scientific reproducibility they provide [7]. However, those cells are often virally immortalized or of uncertain origin and have lost some normal and/or acquired some abnormal properties due to genetic drift [7, 8]. In contrast, primary cells closely resemble the in vivo environment and maintain the differentiated functions of the original cells. Human primary cells offer this superior biological relevance at the cost of limited availability, variability between donors and a finite lifespan [9]. In the context of sepsis research, it has been shown that primary PTEC have unique responses to different strains of lipopolysaccharide (LPS) and this response profile differs significantly from that of the commonly used Human Kidney-2 (HK-2) cell line [8]. Hence, data from experimental studies with human primary PTEC provide relevant insight in SA-AKI pathophysiology that cannot be obtained using immortalized cell lines. The main purpose of this review was to identify and investigate experiments, where human primary PTEC have been applied in studies of sepsis-related factors. Moreover, present an overview of the current research landscape and highlight areas which need further attention. A scoping review was selected as the most suitable methodology for this review due to its effectiveness in addressing broad research questions [10].

## Methods

Our reported items adhered to the Preferred Reporting Items for Systematic Reviews and Meta-Analyses extension for scoping reviews (PRISMA–ScR) checklist [10].

### Search strategy

The search methodology used a building block strategy with Boolean operators. A comprehensive literature search strategy was developed with the participation of all authors, encompassing the period from 1946 to 25th September 2024. The following databases were searched: Ovid MEDLINE(R) and Epub Ahead of Print, In-Process, In-Data-Review & Other Non-Indexed Citations, Daily and Versions, Ovid Embase Classic + Embase, and Clarivate^™^ (Web of Science^™^)^©^ Clarivate 2024^™^. No automatic filtering option was applied. In addition, a manual examination of the reference lists of the retrieved eligible articles was conducted.

**Fig. 1:**
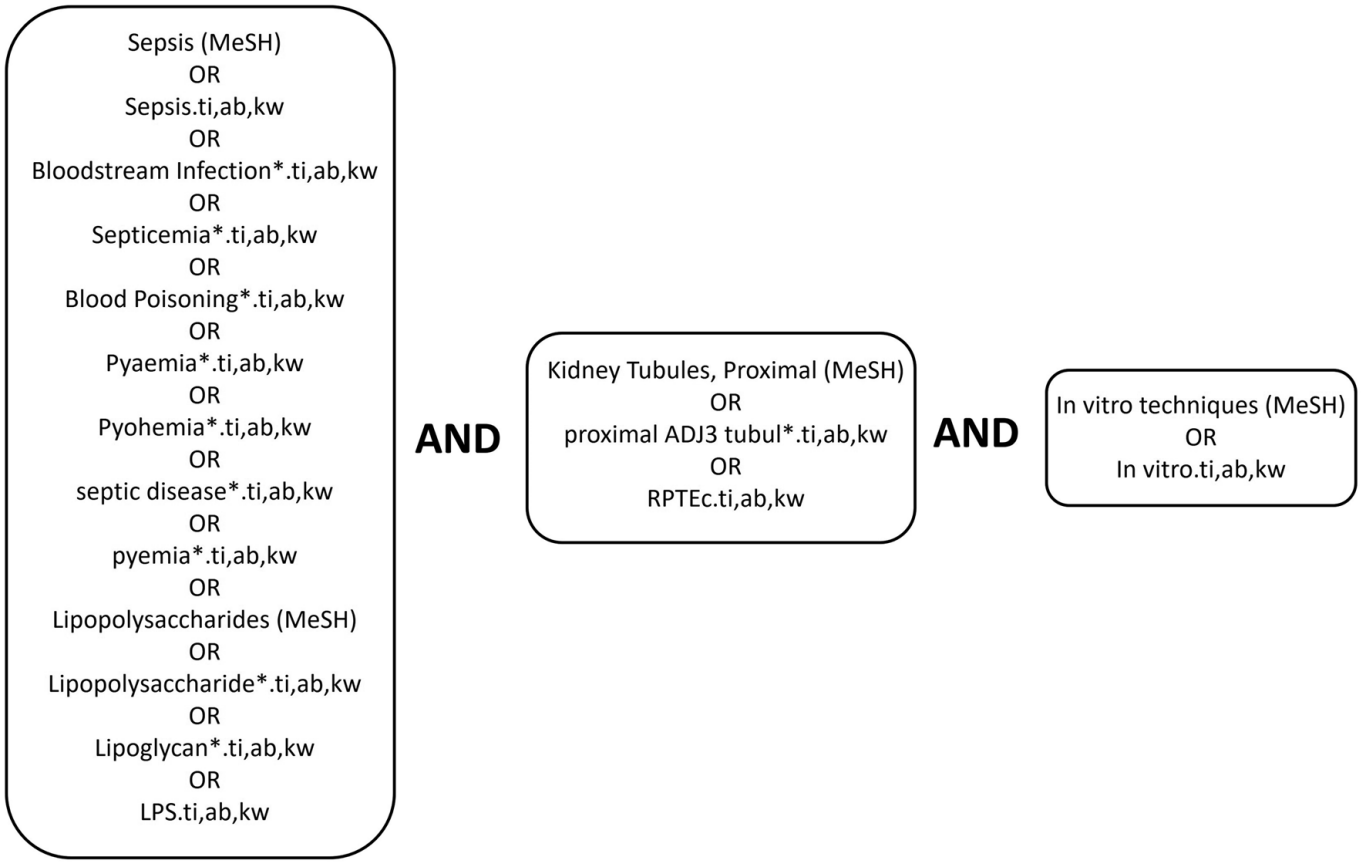
Ovid MEDLINE search strategy

For the human filter applied in Ovid MEDLINE, we used the search syntax from the Cochrane Handbook’s Technical Supplement to Chapter 4 [11]. For Ovid Embase Classic + Embase, we employed a filter derived from Embase: Excerpta Medica Database Guide—Limits: Humans only. For the human filter applied to our Web of Science search, we implemented the filter as described by van der Mierden et al. [12]. The search strategy was in accordance with PRISMA–ScR guidelines [10]. We used the controlled vocabulary of Medical Subject Headings (MeSH) from MEDLINE and the Emtree thesaurus from EMBASE, where applicable. Furthermore, free-text searches of the titles, abstracts, and keywords were conducted in all three databases. We also scanned the reference lists of the included articles for references of interest (citation search/manual search). Complete search strategy from Ovid MEDLINE is provided in Fig. 1.

### Screening, study selection and eligibility criteria

**Fig. 2:**
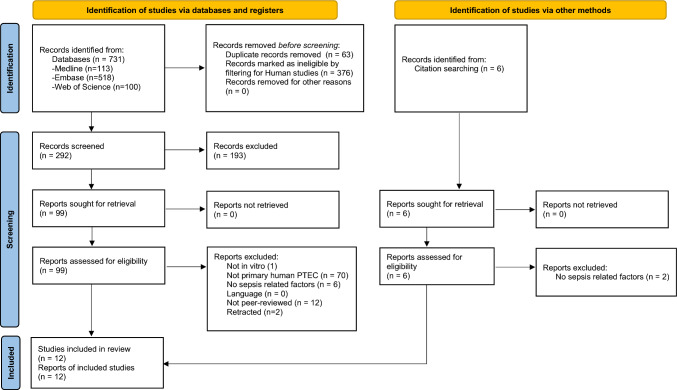
This figure is based on the PRISMA 2020 flow diagram template, which is distributed in accordance with the terms of the Creative Commons Attribution license [28]

All references were exported to EndNote (version 9.7.4; Thomson Reuters, Toronto, ON, Canada), where duplicates were removed. Titles and abstracts from all retrieved references were independently screened by a pair of authors who were blinded to each other’s results. The inclusion criteria are provided in Table 1. Articles that did not meet these criteria were also excluded. Three authors working in pairs subsequently evaluated the full-text articles of the remaining relevant publications for eligibility using the criteria. Conflicts were resolved by consensus and discussion with the third author when necessary. 292 references were screened (Fig. 2). The reference lists of the included articles were screened, and additional references of potential interest underwent the same procedure.

**Table 1:** Inclusion criteria

Study type	In vitro experimental study
Population	Human primary proximal renal tubular epithelial cells
Exposure	Sepsis-related factors, such as lipopolysaccharides, endotoxins, or cytokines
Language	Articles published in English, Swedish, Norwegian, or Danish
Peer-review	Only peer-reviewed articles were included

### Data extraction

Data from the included studies were extracted using a standardized template, capturing key information, such as study objectives, experimental stimuli, cell sources and main findings related to proximal PTEC inflammatory responses. This facilitated the identification of common themes, methodological trends, and research gaps.

## Results

The results of this scoping literature review are included in a PRISMA flow diagram (Fig. 2). A total of 292 abstracts were screened, but only twelve studies fulfilled the inclusion criteria following examination of all retrieved full text articles and the reference lists of the included articles (Fig. 2). Hence, this scoping review encompassed twelve in vitro experimental studies that collectively examined various biological reactions and signaling cascades pertaining to human primary PTEC in the context of sepsis-related factors. Most retrieved articles were excluded, because in vitro experiments had been conducted exclusively with immortalized cell lines (Fig. 2). Data analysis revealed significant heterogeneity in experimental designs and methodological approaches, highlighting both the complexity of human primary PTEC and the critical need for standardization in this research domain. Table 2 provides a summary of the key characteristics of the included articles.

**Table 2:** Summarized key characteristics of included articles

Author. *Title*, Year of publication	Cell origin	Characterization of PTEC phenotype	Summarized PTEC specific objectives	Experimental stimuli	Main PTEC related findings and conclusions
Yard et al. [20] *IL-1α stimulated TNFα production by cultured human proximal tubular epithelial cells*, 1992	Healthy tissue obtained from the non-involved pole during tumor nephrectomy	Characterization of PTEC was performed using various monoclonal antibodies, directed against various antigens	Investigate whether cytokines regulate production of TNF by interacting with PTEC	IL-1 alpha (0.01, 0.1, 1 ng/mL), IL-2 (0-500U/mL), IFNG (0-300U/mL). Exposure for 18 h, in addition to a kinetic experiment with various shorter time intervals LPS stimulation was performed and results reported, but the concentration is not provided	A heterogenous basal production of TNF production, ranging from 0 to 390 pg/mL/10^5^ cells. A dose dependent enhanced production of TNF in response to IL-1 alpha. TNF production by stimulation of PTECs to LPS, interleukin 2 and IFNG was performed, without finding a significant change in TNF production
Schmouder et al. [14] In vitro* and *in vivo* interleukin-8 production in human renal cortical epithelia,* 1992	Normal tissue obtained from the non-involved pole during tumor nephrectomy	Monoclonal immunostaining. Assessment of response in cAMP production by hormonal stimulation	Demonstrate PTECs capability of expressing IL-8 mRNA and secrete IL-8 antigen in response to TNF, IL-1 beta and LPS	IL-1 beta (0.2, 2, 20 ng/mL), TNF (2, 20 ng/mL) or LPS (100 ng/mL, 1 μg/mL) for 1, 2, 4, 8 or 24 h	Primary PTECs responded to the introduction of each of the proinflammatory agents by a dose dependent expression of IL-8 mRNA and secretion of antigenic IL-8 peptide. This proved that the cells could respond to both primary (LPS) and secondary (IL-1 beta, TNF) inflammatory stimulus in a time and dose dependent manner
Boswell et al. [16] *Interleukin 6 Production by Human Proximal Tubular Epithelial Cells *In Vitro*: Analysis of the Effects of Interleukin-1α (IL-1α) and Other Cytokines*, 1994	Healthy tissue obtained from the non-involved pole during tumor nephrectomy Also obtained from biopsy tissue pretransplants and from allografts unsuitable for transplantation	Immunofluorescence using monoclonal antibodies	Examine the kinetics of IL-6 production by PTEC in relationship to stimulatory cytokines	IL-1 alpha (0.01, 0.1, 1, 10 ng/mL), TNF (5, 50, 500 units/mL), IL-2 (20, 100, 500 U/mL), IFNG (10, 100, 1000 U/mL), LPS (1, 10, 100, 1000 μg/mL). Combinations was also used. The cells were stimulated for 24 h	Demonstrated the synthesis and secretion of IL-6 from primary PTECs, and its regulation by IL-1 alpha. IL-1 alpha increased IL-6 production for every separate primary cell line studied, but to variable extent. The study did not find a significant alteration in production of IL-6 by PTECs in response to LPS, IFNG, IL-2 or TNF stimuli
McLay et al. [19] *Nitric oxide production by human proximal tubular cells: A novel immunomodulatory mechanism?,* 1994	Normal pole of human tumor nephrectomy specimens	cAMP response to hormonal stimuli, as well as morphology, dome formation characteristics and maintenance of PTEC typical transport mechanisms	Determine whether PTEC could produce NO in response to stimulation from cytokines	IL-1 beta (10 μg/mL), TNF (10 ng/mL), IFNG (100 μg/mL). The cytokines were applied alone or in combination. The cells were incubated for 24 h	Stimulation from TNF, IFNG and IL-1 beta alone did not induce a production of NO. PTECs had a significant increase in secretion of NO in response to the combination of IL-1 beta and IFNG stimulus, with an additive effect on NO production when co-stimulated with TNF
Biancone et al. [17] *Escherichia coli porin induces proinflammatory alterations in renal tubular cells, *1997	Non-neoplastic kidney tissue from donors undergoing tumor nephrectomy	Immunohistochemistry and cAMP production as response to hormonal stimuli	Investigate the effect of *E. coli* porin on PTECs	36-kD porin purified from *E. coli* at 0.1, 0.5, 1. 5, 10 and 20 μg/mL for up to 24 h	Porin was found to induce a rapid and sustained calcium influx at 10 μg/mL as well as changes in PTEC cytoskeleton consistent with depolymerization of actin Release of TNF, IL-6 and IL-8 was observed at the same porin concentration At the same time, this dose was found to be nonlytic as PTEC viability remained above 95%. A significant reduction in cell viability was only observed in concentrations > 50 μg/mL (the exact dosages is not mentioned in the methods sections and elsewhere)
Krüger et al. [18] *Interleukin-8 Secretion of Cortical Tubular Epithelial Cells Is Directed to the Basolateral Environment and Is Not Enhanced by Apical Exposure to Escherichia coli*, 2000	Normal renal tissue was obtained from tumor nephrectomy	Purity and proximal tubular origin were ensured by immunohistochemistry and electron microscopy	Test whether basolateral IL-8 secretion in PTEC differs from luminal secretion. Investigate if the potential basolateral directed IL-8 secretion could be stimulated by *E. coli* virulence factors	Various *E. coli* mutants (10^8^/mL), S. fimbriae isolates (1 mg/mL), *E. coli* LPS (1 mg/mL) and IL-1 alpha (1 ng/mL) for 24 or 48 h Stimulation was specifically performed at the apical side of the PTECs	Incubation with IL-1 alpha resulted in a significant increase in basolateral IL-8 production, while no increase was seen following stimulation by *E. coli* mutants, S. fimbriae or *E. coli* LPS compared to controls
Glynne et al. [15] *Coexpressed Nitric Oxide Synthase and Apical β*_*1*_* Integrins Influence Tubule Cell Adhesion after Cytokine-Induced Injury*, 2001	Normal renal tissue from fresh tumor nephrectomy specimens	Characterized morphologically, immunochemically (cytokeratin staining) and by hormone responsiveness	Determine the effects of combination of inflammatory cytokines observed in sepsis on human PTEC in the absence of complicating hemodynamic factors	TNF (10 ng/mL), IL-1 alpha (10 ng/mL), and IFNG (200 U/mL) alone and in combinations. Stimulation was performed up to 24 h	Dramatical morphological alterations already at 2 h with elongation and extensive filopodal processes. Marked disruption of basal actin stress fibers. Stimulation for 24 h provided the greatest NO release. Excessive production of NO resulted in cytoskeletal disorganization and cell detachment, suggesting that inhibition of NO may protect PTEC from detachment and contribute to the maintenance of tubular integrity during sepsis-induced renal injury
Nitschke et al. [13] *Bactericidal Activity of Renal Tubular Cells: The Putative Role of Human ß-Defensins*, 2002	Uninvolved pole of kidney removed for tumor nephrectomy	Refer to a separate source for methods of obtaining PTEC, but Nitschke et al. do not specify that they characterized the phenotype of their PTECs	Investigate the antimicrobial activity of PTECs in vitro in response to various inflammatory stimuli	IL-1 alpha (30 ng/mL), IL-6 (100 U/mL), TNF (100 ng/mL), LPS (2 μg/mL) or *E. coli* (1 mL OD 0.1 bacteria in 5 mL culture medium) for 16–48 h	Strong bactericidal activity against *E. coli* and Klebsiella pneumoniae was demonstrated in cell culture supernatants from PTECs The researchers found no significant difference in antibacterial activity between the PTECs stimulated by the cytokines, LPS and bacteria and unstimulated PTECs, which suggests that constitutively expressed factors were responsible for this finding
Ionin et al. [24] *Staphylococcal enterotoxin B causes differential expression of Rnd3 and RhoA in renal proximal tubule epithelial cells while inducing actin stress fiber assembly and apoptosis*, 2008	Obtained via vendor (Clonetics Corp., Walkersville, MD). Originally PTECs from a 34-year-old African American male donor	Information not provided	To test the hypothesis that SEB induce dysregulation of vascular tone through PTEC cells	50 µg/mL of SEB from Staphylococcus aureus strain 10–275 for 2, 6, 12, 24, 48 and 72 h	Time dependent SEB-induced differential gene expression, with down-regulation of Rnd3 and upregulation of RhoA. Furthermore, the authors found that PTECs incubated for 24 h had a marked increased strain intensity, demonstrating different intracellular actin distribution compared to controls. Lastly, SEB was demonstrated to increase apoptosis, with more than 50% of PTEC cells being apoptotic at 24 h. The authors summarize the results as indications of SEB induced actin stress fiber formation as a contributor to cell death
Mendis et al. [23] *Effect of 5-Lipoxygenase Inhibitor MK591 on Early Molecular and Signaling Events Induced by Staphylococcal Enterotoxin B in Human Peripheral Blood Mononuclear Cells,* 2008	Obtained via vendor (Clonetics Corp., Walkersville, MD). Originally PTECs from a 34-year-old African American male donor	Information not provided	Evaluate multiple signaling cascades induced by SEB and target pathway interconnector 5-LO to inhibit unwanted cellular activities	100 ng/mL SEB from Staphylococcus aureus strain 10–275 for 5 min	MK591 effectively inhibited proliferation of SEB stimulated PTEC by 33%, while SB203580 showed no inhibitory effect on proliferation
Hammamieh et al. [21] *Characterization of the Interaction of Staphylococcal Enterotoxin B with CD1d Expressed in Human Renal Proximal Tubule Epithelial Cells*, 2015	PTECs were purchased from Lonza (Frederick, MD). No further details on the origins of the cells were provided	Information not provided	Understand the renal response to SEB shock, focusing on the interaction of SEB with CD1d in PTECs	100 µg/mL SEB at 15, 30, 60 and 120 min. SEB vendor is specified, but not the staphylococcal strain of origin	Time sensitive co-localization of SEB with CD1d expressed on PTEC surface, with a direct conjugation of SEB to CD1d. The researchers found that inhibiting CD1d negatively impacts the binding of SEB to PTECs, which suggested a functional role for CD1d in antigen recognition, cleansing and sequestration. This suggested that the interaction between CD1d and SEB facilitated a surge in cytokines due to CD1d and natural killer T cell crosstalk
Ding et al. [22] *CSTMP Exerts Anti-Inflammatory Effects on LPS-Induced Human Renal Proximal Tubular Epithelial Cells by Inhibiting TLR4-Mediated NF-κB Pathways*, 2016	Purchased via commercial provider (FMG-BIO, Sciencell, Shanghai, China). No further details on the origins of the cells were provided	Identity and purity of the cells were confirmed by staining for γ-glutamyl transferase	Investigate the cytoprotective effects of CSTMP on PTEC	LPS (1 μg/mL) for 6 or 24 h. The cells were pretreated with different concentrations of CSTMP from 0 to 100 μgM for 1 h	Inhibition of the TLR4 pathway using CSTMP significantly reduced NF-κB activation, thereby decreasing pro-inflammatory cytokine production and attenuating cellular damage. These findings suggested that the TLR4/NF-κB pathway may represent a therapeutic target for mitigating renal inflammation in sepsis

### Cell culture origin

Eight studies used human primary PTEC isolated by the researcher group [13–20]. All eight of these obtained their primary PTECs by sampling from healthy tissue during tumor nephrectomy. One of these studies also obtained primary PTECs from biopsy tissue pretransplants and from allografts unsuitable for transplantation [16]. Seven of the studies specified how they characterized the phenotypes of the PTECs [14–20]. The four remaining studies used PTEC purchased from specified commercially available providers [21–24]. Of these four, only two studies provided further details on the origins of the primary PTECs [23, 24].

### E. coli porine

Biancone et al. [17] studied PTEC response to Escherichia coli (*E. coli*) porine. The researchers found a significant reduction in cell viability for high concentrations after 1 h of treatment. Porin in lower concentrations induced changes in PTEC cytoskeleton consistent with depolymerization of actin. The researchers point to the critical role adherence junctions has in maintaining cell polarity in PTECs, suggesting that the phenomenon may be due to loss of polarity and dysfunction of PTECs in Gram-negative sepsis.

### Staphylococcus enterotoxin

Three studies evaluated the effects of Staphylococcal enterotoxin B (SEB) on PTECs.

Hammamieh et al. exposed PTECs to SEB at 100 µg/mL from 15 min to 2 h[21]. The researchers studied PTEC cluster of differentiation 1d glycoprotein (CD1d) interaction with SEB. Hammamieh et al. [21] found time sensitive co-localization of SEB with CD1d expressed on PTEC surface and they demonstrated the direct conjugation of SEB to CD1d. Furthermore, the researchers found that inhibiting CD1d negatively impacts the binding of SEB to PTECs, which suggested a functional role for CD1d in antigen recognition, cleansing and sequestration. This implies that the interaction between CD1d and SEB facilitated a surge in cytokines due to CD1d and natural killer T cell crosstalk.

Mendis et al. exposed PTEC to SEB from Staphylococcus aureus strain 10–275 at 100 ng/mL for 5 min [23]. SEB induces a pro-inflammatory signaling cascade leading to upregulation of inflammatory mediators. The researchers investigated the effects of two known inhibitors of SEB mediated tumor necrosis factor (TNF) upregulation, MK591 and SB203580, on SEB treated PTECs. MK591 effectively inhibited PTEC proliferation by 33%, while SB203580 showed no inhibitory effect.

Ionin et al. [24] exposed PTEC to SEB from Staphylococcus aureus strain 10–275 to examine the PTEC for morphological changes and gene expression. The cells were exposed with 50 µg/mL of SEB for 2–72 h. The authors found a time dependent SEB-induced differential gene expression. Furthermore, the authors found that PTECs incubated for 24 h demonstrated different intracellular actin distribution compared to controls. Finally, SEB was demonstrated to increase apoptosis, with more than 50% of PTEC cells being apoptotic at 24 h.

### Cytokines

Five studies exposed PTECs to cytokines alone. Yard et al. studied Interleukin-1 alpha (IL-1 alpha) stimulated TNF production by PTEC [20]. The PTECs were exposed to various concentrations of IL-1 alpha (0.01, 0.1, 1 ng/mL). There was a basal production of TNF production ranging from 0 to 390 pg/mL/10^5^ cells. The authors found a dose dependent enhanced production of TNF in response to IL-1 alpha. Furthermore, the authors examined TNF production by a similar stimulation of PTECs to interleukin 2 and Interferon gamma (IFNG) without finding a significant change in TNF production.

McLay et al. investigated if PTEC could produce nitric oxide (NO) in response to stimulation from cytokines [19]. The PTECs were incubated for 24 h with the proinflammatory cytokines Interleukin-1 beta (IL-1 beta) (10 μg/mL), TNF (10 ng/mL), IFNG (100 μg/mL). The cytokines were applied alone or in combination. The authors found that stimulation from TNF, IFNG and IL-1 beta alone did not induce a production of NO. PTECs had a significant increase in secretion of NO in response to the combination of IL-1 beta and IFNG stimulus, with an additive effect on NO production when co-stimulated with TNF. The authors concluded that the production of NO by PTEC is regulated by cytokines.

Glynne et al. exposed cells to human TNF (10 ng/mL), IL-1 alpha (10 ng/mL), and IFNG (200U/mL), arguing that these levels reflected levels observed in patients diagnosed with sepsis [15]. This study investigated the role of NO in mediating PTEC detachment and apoptosis under cytokine-induced stress. The results indicated that NO plays a crucial role in modulating cell shedding and cytoskeletal alteration [15]. Excessive production of NO resulted in cytoskeletal disorganization and cell detachment, suggesting that inhibition of NO may protect PTEC from detachment and contribute to the maintenance of tubular integrity during sepsis-induced renal injury.

Boswell et al. [16] studied interleukin 6 (IL-6) production in response to IL-1 alpha, TNF, interleukin 2 (IL-2), IFNG or LPS at varying dosages, within the range attainable in vivo. The study demonstrated the synthesis and secretion of IL-6 from primary PTECs, and its regulation by IL-1 alpha. The researchers found that IL-1 alpha increased IL-6 production for every separate primary cell line studied, but to variable extent. The study did not find a significant alteration in production of IL-6 by PTECs in response to other stimuli.

Schmouder et al. [14] studied expression of Interleukin 8 (IL-8) mRNA by proinflammatory stimulation by Interleukin-1 beta (IL-1 beta), TNF or LPS at varying concentrations and for varying lengths of time. The study revealed that primary PTECs responded to the introduction of these proinflammatory agents by a dose dependent expression of IL-8 mRNA and secretion of antigenic IL-8 peptide. Furthermore, this proved that the cells could respond to both primary (LPS) and secondary (IL-1 beta or TNF) inflammatory stimulus in a time and dose dependent manner.

### Cytokines, LPS and bacteria

Nitschke et al. studied the host defense and barrier function of PTECs [13]. The authors hypothesized that the bacteria could directly produce antimicrobial substances, arguing that the presence of the antimicrobial peptide human beta-defensin (hbd-1) had already been proven in renal tissue. Nitschke et al. exposed PTECs to cytokines (IL-1 alpha (30 ng/mL), IL-6 (100 U/mL), TNF (100 ng/mL), LPS (2 μg/mL) and *E. coli*) for 16–48 h [13]. The authors found that PTECs exerted strong antimicrobial activity in vitro. The researchers found no significant difference in antibacterial activity between the PTECs stimulated by the cytokines, LPS and bacteria and unstimulated PTECs.

Krüger et al. studied the basolateral secretion of Interleukin 8 (IL-8) to virulence factors of *E. coli* [18]. PTECs were stimulated on the apical side by various *E. coli* mutants (10^8^/mL), S fimbriae isolates (1 mg/mL), *E. coli* LPS (1 mg/mL) and IL-1 alpha (1 ng/mL) for 24 or 48 h. Incubation with IL-1 alpha resulted in an significant increase in basolateral IL-8 production, while no increase was seen following stimulation by other stimuli compared to controls.

### LPS

Ding et al. [22] exposed the PTECs to LPS, studying the cytoprotective effects of (E)-2-(2-chlorostyryl)-3,5,6-trimethylpyrazine (CSTMP). The researchers exposed cells to LPS at 1 μg/mL for 6 or 24 h. The cells were pretreated with different concentrations of CSTMP. The study demonstrated that the inhibition of the TLR4 pathway using CSTMP significantly reduced NF-κB activation. These findings suggested that the TLR4/NF-κB pathway may represent a therapeutic target for mitigating renal inflammation in sepsis.

## Discussion

This scoping review provides a comprehensive list of publications in which primary PTECs have been utilized for experiments relevant to SA-AKI.

Few articles fulfilled the inclusion criteria. Twelve peer reviewed papers were included, of which, only two were published after 2010. Furthermore, an overwhelming majority of the screened articles were excluded, because experiments were conducted with immortalized cell lines. The human kidney-2 (HK-2) cells were by far the most frequently used. Although primary PTECs more accurately reflect in vivo conditions, they are constrained by availability and inter-donor variability [7, 9]. Conversely, immortalized cell lines provide consistency, but may not fully recapitulate primary cell physiology, often exhibiting altered gene expression profiles and phenotypic changes [25]. HK-2 cells arise from primary human proximal tubular cells exposed to a recombinant retrovirus. This cell line express human papillomavirus E6 and E7 genes, making the cell line able to grow continuously in serum free media and be utilized for in vitro experiments over time [26]. These oncogenes may also induce other hallmarks of cancer cells [27]. Although in vitro studies with immortalized cell lines offer ease of use, low costs, and high reproducibility, the validity of their data has been questioned, primarily because these cell lines are phenotypically different from human primary PTECs [7–9, 25]. Hence, in vitro experiments specifically carried out with human primary PTEC cultures are clearly more relevant for the study of SA-AKI and may provide relevant data that are not obtainable in any other way.

Data analyses of the included articles revealed that PTECs play an active role in the inflammatory and metabolic disturbances observed in patients with SA-AKI [3]. PTECs role in SA-AKI is multifaceted, involving both deleterious processes, such as pro-inflammatory cytokine production and protective mechanisms aiming at restoring cellular homeostasis [13, 14, 16, 21]. The pivotal role of PTECs in SA-AKI pathogenesis presents both challenges and opportunities. Although PTECs contribute to kidney damage through inflammatory and metabolic disturbances, mechanisms involving PTECs might also serve as potential therapeutic targets [15, 22, 23]. While the included articles provide relevant insights into sepsis and SA-AKI, the limited use of human primary PTEC results in substantial research gaps in this field. Conducting future experiments with primary PTECs have the potential to fill these gaps and advance the understanding of SA-AKI pathophysiology, prevention and treatment.

A significant scientific challenge identified in this review is the heterogeneity of experimental models applied in the various studies. The studies that utilized LPS as a proinflammatory stimulus applied it at varying doses and length of exposure [13, 14, 16, 18, 22]. Hammamieh, Ionin and Mendis all exposed PTECs to SEB, but at varying doses and length of exposure [21, 23, 24]. Cytokines were applied in several studies, sometimes at fixed dosages and other times at varying levels, with considerable differences in both dosage and length of exposure across studies [13–16, 19, 20]. The utilization of heterogeneous PTEC sources and the variety of experimental conditions, impede the comparability of data. Furthermore, limited information about commercially provided primary PTECs are also a concern. We believe the topic to be unsuitable for a systematic review due to the limited amount of existing literature and the heterogeneity identified in this scoping review.

To enhance reproducibility and facilitate data aggregation across studies with primary PTECs, the standardization of experimental protocols, including cell culture conditions and sepsis-related stimuli, may prove useful. Unfortunately, the current literature on human primary PTECs does not provide sufficient basis for specific proposals for standardization due to the extensive methodological heterogeneity discovered in this scoping review. Therefore, alternative methodologies beyond literature reviews will be required to develop standardization guidelines.

The use of Boolean operators in conjunction with broad search terms provided a robust literature search, which represents a key strength of this review. A possible limitation to our research is that we have included studies that have created sepsis-like conditions, even when there is no specific intent from the authors to study sepsis or SA-AKI. Our rationale has been that data from these studies could potentially be relevant at elucidating SA-AKI pathophysiology.

## Conclusion

This comprehensive scoping review provides fresh insight into sepsis and SA-AKI pathophysiology by summarizing research, where human primary PTEC has been utilized in experimental conditions that mimic sepsis. It is noteworthy that a thorough literature review spanning 80 years uncovered such remarkably few studies using primary human PTEC as an inflammatory model system. Although human primary PTEC more closely resembles the in vivo environment in human kidneys, their use in sepsis and SA-AKI research remains limited, leading to substantial research gaps in this field. In addition, there is significant heterogeneity in the methodologies employed across existing studies. Standardizing and expanding the use of primary PTECs in future in vitro research could be pivotal for unraveling novel and relevant insights into the pathophysiology of SA-AKI.

## Declarations

### Human and animal participants

This article does not contain any studies with human participants or animals performed by any of the authors.
